# Biomarkers for immune thrombocytopenia

**DOI:** 10.1186/s40364-015-0045-0

**Published:** 2015-07-16

**Authors:** Lingjia Yu, Chunmei Zhang, Liping Zhang, Yongyu Shi, Xuebin Ji

**Affiliations:** Department of Hematology, Qilu Hospital, Shandong University, 107 West Wenhua Rd, Jinan, Shandong 250012 PR China; Department of Hematology, the central Hospital of TaiAn, TaiAn, PR China; Jinan Stomatological Hospital, Jinan, PR China; Institute of Immunology, School of Medicine, Shandong University, Jinan, PR China

**Keywords:** Immune thrombocytopenia, Biomarker, T cell, B cell, Pathogenesis

## Abstract

Immune thrombocytopenia is an autoimmune disease with abnormal biomarkers. Immune thrombocytopenia pathogenesis is a complicated process in which the patient’s immune system is activated by platelet autoantigens resulting in immune mediated platelet destruction or suppression of platelet production. The autoantibodies produced by autoreactive B cells against self antigens are considered to play a crucial role. In addition, biomarkers such as transforming growth factor-beta1,Toll-like receptors,T helper 1 andT helper 2 cytokine bias, Notch signaling and abnormal biomarker in megakaryocyte maturation are involved in the pathogenesis of this disease. With the genomewide association study on immune thrombocytopenia, more biomarkers will be founded in the future. They may provides a theoretical basis for the mechanism and treatment of immune thrombocytopenia.

## Introduction

Immune thrombocytopenia (ITP) is an autoimmune bleeding disorder in which the patient’s immune system is activated by platelet autoantigens resulting in immune-mediated platelet destruction and/or suppression of platelet production with the clinical manifestations of oral gingival and skin mucous membrane bleeding.

Biomarkers are very important to the pathogenesis of disease. Many abnormal immune biomarkers play crucial roles in ITP pathogenesis [1]. The abnormal B, T cell and some new biomarkers in ITP were introduced. It may help people to know the complicated pathogenesis of ITP.

### Abnormal B cell immune biomarkers

ITP is an autoimmune disease. Glycoprotein (GP) specific autoantibodies (e.g., GPVI, GPIb/IX, GPIIb/IIIa autoantibodies) can be detected in most ITP patients’ plasma or platelet eluates by modified monoclonal antibody immobilization of platelet antigen (MAIPA) assay, which bind to the specific glycoproteins by an antigen-binding fragment (Fab) and then activate the mononuclear-macrophage or complement system [2, 3]. The autoantibodies produced by autoreactive B cells against self-antigens, specifically immunoglobulin G (IgG) antibodies against GPIIb/IIIa and/or GPIb/IX, are considered to play a crucial role in the plaletet destruction [4].

Approximately 75 % of platelet autoantigens are localized to either the platelet GP IIb/IIIa or GP Ib/IX complex. Inhibition of the binding of autoantibodies from several ITP patients by either another ITP autoantibody or by a monoclonal anti-GPIIb/IIIa antibody suggests that the antigenic repertoire in chronic ITP may be limited. Many patients may produce multiple antibodies. Once produced, antiplate autoantibodies may either bind to platelets, causing their destruction by either phagocytosis or possibly complement activation and lysis, or bind to megakaryocytes, resulting in decreased thrombopoiesis. A variety of new investigative techniques including MAIPA assay have localized a few autoantigens to specific regions of the cytoplasmic or extracellular regions of both GPIIb/IIIa and GPIb/IX. These methods that allow incubation of patient serum or plasma with intact platelets (MAIPA and immunobead) have greater sensitivity than techniques in which the patient antibody is tested against previously isolated platelet glycoproteins [5].

Platelets with affiliated IgG are targeted for exploitation by Fc receptor-mediated phagocytic cellular material within the reticuloendothelial system. The expression of FC gamma receptors (FCGR) IIb was decreased in ITP. Lower expression of FCGRIIb may be involved in the pathogenesis of ITP. An increasing in FCGRIIb may be considered as a therapeutic target for human ITP treatment or possibly as a new biomarker for ITP analysis [6].

It was demonstrated decreased expression of FCGRIIb and elevated expression of FCGRIIa and FCGRIII on monocytes in ITP patients before high-dose dexamethasone (HDDXM) therapy. HD-DXM treatment may change the FCGR balance toward the inhibitory FCGRIIb, thus providing new insights into the mechanism of HD-DXM in the treatment of ITP [7].

B-cell activating factor (BAFF; also known as B-lymphocyte stimulator) belonging to the family of tumor necrosis factor (TNF) ligands is critical for the maintenance of normal B-cell development, homeostasis, and autoreactivity [8, 9] and T-cell costimulation [10–12]. BAFF is an important homeostatic cytokine for B cells that is up-regulated during inflammation and links adaptive with innate immunity. BAFF has been shown to enhance the expression of CD19 and mediate the maturation of autoreactive B cells. It is shown that BAFF is elevated in ITP patients with active disease, and excessive BAFF may rescue autoreactive B and T cells from apoptosis [13].

### Abnormal T cell immune biomarkers

The cytokine transforming growth factor-beta1 (TGF-β1) is a central player in maintaining the immune response balance, which belongs to regulator T cell (Treg) cytokines. Tregs play crucial roles in the induction and maintenance of immune tolerance, which are involved in ITP abnormal T cell immunity. It was found that the concentration TGF-β1 was lower in ITP patients [14]. The dominant function of TGF-β1 is to regulate peripheral immune homeostasis [15].

Toll-like receptors (TLRs) are the major molecules involved in the immune regulatory process [16, 17]. TLRs play an important role in defending the host against pathogenic microbes through the induction of inflammatory cytokines and type I interferons. Furthermore, TLRs play roles in shaping pathogen-specific humoral and cellular adaptive immune responses [18]. TLRs have been found to be associated with immune-mediated diseases but it is still not clear whether they play a role in ITP, especially TLR4. CD4+ T-lymphocyte abnormalities, including T helper (Th)17. Th1, Th2, and Treg are considered important in ITP. It was showed that TLR4, CD4+ T cells (Th1, Th17 and Treg cells) and related cytokines may take part in the pathogenesis of ITP. TLR4 may play a role through the TLR4-cytokine-CD4+ T lymphocyte cell pathway [19].

Th1 polarization persists in the autoimmune response found in ITP. TLR7 expression, which also plays an important role in autoimmune diseases, was found increasing in ITP. The findings indicate that TLR7 promotes Th1 polarization and may contribute in ITP pathogenesis [20].

Th1/Th2 cytokine bias may be an important mechanism of ITP. The Th1/Th2 balance is important to normal human immunity. Th1 cells primarily secrete the inflammatory cytokines interleukin (IL)-2, IL-12, interferon-gamma (IFN-γ) and TNF-β. Th2 cells primarily secrete the inflammatory cytokines IL-4, IL-5, IL-6 and IL-10.

ITP is associated with a Th1 type of T helper cytokine response, while that of type Th2 is downregulated. The present study demonstrates that an imbalance of Th1 and Treg cytokines may mediate the pathogenesis of ITP [21]. Uncontrolled Th-1 lymphocyte activation may be an important mechanism of ITP. Treatment with a large dose of dexamethasone, the Th1-type cytokine spectrum returns to the dominant position after treatment with dexamethasone [22]. Shifting the cytokine patterns from Th1 to Th2 may be a potential immunotherapy for ITP.

Cytotoxic T-lymphocytes (CTLs) can induce the lysis of autologous platelets. CTLs-mediated platelet destruction and aberrant cytokine profiles play important roles in the pathogenesis of ITP. Interleukin-27 (IL-27) has pleiotropic immunomodulatory effects. The results suggest that IL-27 can inhibit CTL-mediated platelet destruction by negatively regulating CTL cytotoxicity toward platelets in ITP. IL-27 may have a therapeutic role in treating ITP patients [23].

Notch signaling has been implicated in peripheral T-cell activation and effector cell differentiation. Th17 cells and Treg cells, along with Th1 and Th2 cells, may contribute to the pathogenesis of ITP. The imbalance of Th17/Treg toward Th17 cells has been shown to play an important role in the peripheral immune response. However, the role of Th17/Treg in the pathogenesis of ITP and the effect of Notch signaling on Th17/Treg imbalances still remain uncertain in ITP [24].

To investigate the role of Notch signaling pathway in ITP, the expression of Notch pathway molecules in ITP patients were measured and evaluated their clinical relevance. It was found that Notch1 and Notch3 expression elevated, while Notch2 decreased statistically in ITP patients. The findings suggest that the Notch signaling pathway may be involved in ITP. The blockage of Notch1 pathway may be a promising therapeutic method [25].

### Abnormal biomarker in ITP megakaryocyte maturation

Tumor necrosis factor-related apoptosis-inducing ligand (TRAIL) belongs to the TNF super-family protein biomarkers. TRAIL can promote the apoptosis and maturation of megakaryocytes. It was demonstrated that low expression of TRAIL in megakaryocytes contributed to impaired platelet production in ITP. TRAIL expression on megakaryocytes and the TRAIL concentrations of ITP patients were decreased. Megakaryocyte apoptosis mediated by TRAIL in the plasma of ITP patients may be a potential mechanism by which the megakaryocyte number increases in vitro [26]. It was found that low dose decitabine can promote megakaryocyte maturation and platelet production and enhance TRAIL expression in megakaryocytes in healthy controls and ITP [27].

### New biomarkers in ITP

Genetic sequence variations, such as single nucleotide polymorphisms (SNPs), may have biological roles in modifying the outcome risk of many diseases. Genomewide association study(GWAS) is a new method that can be used to find genetic characteristic SNPs of many complex disease by case–control association analysis [28, 29]. GWAS on ITP may find some genetic SNPs which can be developed to new biomarkers for the clinical practice, individualized diagnosis and treatment of ITP.

## Conclusions

ITP is an autoimmune disease with abnormal immune biomarkers. The autoantibodies produced by autoreactive B cells against self-antigens, specifically antibodies against GPIIb/IIIa and/or GPIb/IX, are considered to play a crucial role in the pathogenesis of ITP. Biomarkers such as TGF-β1,TLRs, Notch signaling, TRAIL,Th1/Th2 cytokine bias are involved in ITP T cell abnormality. With the GWAS on ITP, more biomarkers will be founded in the future. They may provides a theoretical basis for the mechanism and treatment of ITP.

## Abbreviations

ITP: immune thrombocytopenia
MAIPA: modified monoclonal antibody immobilization of platelet antigen
Treg: T regulatory cell
FCGR: FC gamma receptors
Th: T helper
TLR: toll-like receptors
IFN-γ: interferon-gamma
TGF-β: transforming growth factor-beta
IL: interleukin
TNF-β: tumor necrosis factor-beta
GP: glycoprotein
TRAIL: tumor necrosis factor-related apoptosis-inducing ligand
GWAS: genomewide association study
